# 3D assessment of root resorption and soft tissue adaptations in surgery-first vs. early orthognathic surgery: a randomized clinical trial

**DOI:** 10.1007/s00784-026-06846-3

**Published:** 2026-03-30

**Authors:** Halime Saraç Kale, Gökhan Çoban, Ahmet Emin Demirbaş, Emrah Soylu

**Affiliations:** 1 Department of Orthodontics, Faculty of Dentistry, B Block Floor:3, Erciyes University, 38039, Melikgazi Kayseri, Türkiye; 2 Department of Oral and Maxillofacial Surgery, Faculty of Dentistry, Erciyes University, 38039, Melikgazi Kayseri, Türkiye

**Keywords:** Three-dimensional diagnosis and treatment planning, Soft tissue, Periodontics, Class III orthognathic surgery

## Abstract

**Objective:**

This study evaluated the effects of two orthognathic surgery-timing approaches on hard and soft tissues in skeletal Class III patients undergoing bimaxillary orthognathic surgery using cone beam computed tomography (CBCT) and stereophotogrammetry.

**Methods:**

Patients undergoing non-extraction surgery were randomly assigned to either a surgery-first (SF) group (*n* = 12; 19.91 ± 2.60 years) or a surgery-early (SE) group (*n* = 12; 22.50 ± 2.90 years) for a total of 24 patients. Maxillary advancement was 3.64 ± 1.50 mm in the SF group and 3.47 ± 2.77 mm in the SE group, and mandibular setback was 2.25 ± 1.32 mm and 1.20 ± 1.75 mm, respectively. Millimetric (length, dehiscence, and fenestration), volumetric measurements, and soft tissue anthropometric assessments were collected using three-dimensional (3D) records obtained before treatment and after debonding.

**Results:**

Following orthognathic surgery, a significant reduction in root length and volume measurements, and a significant increase in dehiscence were observed within the groups. Additionally, incisor teeth exhibited greater root resorption compared to other teeth. However, no significant differences were found between the groups regarding changes in hard or soft tissues.

**Conclusions:**

These findings suggest that changes in root length, alveolar bone, and soft tissues are similar in the SF and SE approaches, indicating that these changes occur independently of the surgical timing in comparable patients.

**The trial registration number (TRN):**

is NCT06873451 (Date of registration: 13.02.2025; retrospectively registered).

**Supplementary Information:**

The online version contains supplementary material available at 10.1007/s00784-026-06846-3.

## Introduction

Skeletal Class III malocclusions, which arise due to genetic and environmental factors, are among the most aesthetically and functionally concerning dentofacial anomalies. [1] Orthognathic surgery, used to correct severe skeletal and/or dental discrepancies that hinder functionality and/or social acceptability, improves skeletal structure and influences mastication, speech, and facial aesthetics via changes in the surrounding soft tissues. [2–4] In orthognathic surgery, the most commonly performed surgical procedure for the maxilla is the Le Fort I osteotomy and for the mandible, the bilateral sagittal split ramus osteotomy. [5] It has been reported that impaired collateral circulation and reduced blood flow after surgery may lead to necrosis of bone and soft tissues, devitalization of the teeth, and periodontal loss. [6] Therefore, detecting and monitoring root resorption during the treatment process is critically important for the long-term prognosis of the teeth. [7–9]

The surgery-first (SF) approach, introduced in the 1960s [10], creates transitional occlusion using osteotomies. [11] With this technique, the patient’s primary concern, aesthetic discomfort, is addressed from the outset, which enhances the patient’s compliance with postoperative orthodontics. [12] Another advantage is the reduction in overall treatment time, associated with the accelerated bone remodeling and the reshaping process due to an increase in osteoclastic activity and metabolic changes in the dentoalveolus in the osteotomized area, termed the regional acceleratory phenomenon (RAP). [11, 13]

In the surgery-early (SE) approach, although patients may request an immediate aesthetic change [14], orthognathic surgery can be performed after partial orthodontic preparation due to reasons such as midline deviation or mild crowding. This approach helps eliminate negative factors including postoperative instability or occlusal disharmony (e.g., insufficient overbite or loss of canine width), and it strengthens postoperative immobilization [15].

The literature examined treatment duration and patient satisfaction in both the SF and SE approaches, with aesthetic improvement identified as the primary motivator for treatment. [13, 16] In the present study, the null hypothesis that there would be no significant difference between the SF and SE approaches in terms of root resorption, volume, or soft tissue changes was tested. No studies comparing these approaches using three-dimensional (3D) hard and soft tissue measurements were found. Therefore, the primary objective of the present study was to evaluate volumetric changes in tooth roots, whereas the secondary objectives included assessing changes in root length, dehiscence, fenestration, and soft tissue anthropometric measurements.

## Materials and methods

### Study design

Ethical approval for this prospective, randomized, clinical trial was obtained from the Clinical Research Ethics Committee of Erciyes University (Decision No: 2023/655, Date: 04.10.2023) and the Türkiye Medicines and Medical Devices Agency (TİTCK) (No.23-AKD-311). The present study has also been registered on ClinicalTrials.gov with the number NCT06873451. A power analysis was conducted using a paired t-test (matched pairs) for a one-sided alternative hypothesis with G*Power Software (version 3.1.9.7; Universität Düsseldorf, Germany). The a priori sample size calculation was based on the primary outcome variable, the volumetric change in the apical third of the mandibular central incisor root. The effect size was estimated based on a previously published 3D CBCT study evaluating root resorption in skeletal Class III patients, using the pre- and post-treatment mandibular central incisor root volume at the apical third (BVA, mm³) values (19.94 ± 5.18 mm³ pre-treatment and 15.04 ± 4.03 mm³ post-treatment). [17] Based on these values, the calculated effect size (dz) was 1.04. Considering a significance level (α) of 0.05 and a statistical power (1 - β) of 0.90, the required sample size was calculated as 10 patients per group. To compensate for possible dropouts, 12 patients were included in each group, resulting in a total sample size of 24 patients. After being informed about the treatment and study, written informed consent was obtained from participants.

### Patient selection

Patients seeking orthognathic surgical treatment at the Department of Orthodontics, Faculty of Dentistry, Erciyes University and candidates for non-extraction surgery were randomly assigned to either the SF or the SE group. Participants were allocated to groups via a computer-assisted randomization program (http://random-allocation-software.software.informer.com/2.0/). [18] Randomization was performed using a 1:1 block randomization scheme, and the randomization sequence was generated before the initiation of the study. Allocation concealment was maintained until the time of group assignment. All measurements were performed by the same researcher (HSK), who conducted the clinical procedures. Statistical analyses were performed by an independent statistician who was blinded to group allocation and treatment protocols.

The mean age of the participants in the SF group was 19.91 ± 2.60 years (5 females, 7 males), while the mean age in the SE group was 22.50 ± 2.90 years (9 females, 3 males). Body mass index (BMI) values were recorded at baseline for all participants. All included patients had BMIs within the normal range (18–25 kg/m²). The mean BMI was 21.34 ± 2.66 kg/m² in the SF group and 22.30 ± 2.43 kg/m² in the SE group.

Inclusion criteria comprised patients over 18 years of age with skeletal Class III malocclusion who required bilateral orthognathic surgery and had minimal crowding (0–3 mm), complete 3D records, no dental caries, good oral hygiene, and healthy periodontal tissues. Good oral hygiene and healthy periodontal tissues were assessed clinically at baseline during routine periodontal examination. During the study period, no severe pain, excessive gingival bleeding, gingival recession, or tooth mobility was observed, and the overall gingival condition was assessed by clinical inspection. Maxillary and mandibular crowding were assessed on pre-treatment dental casts using arch length discrepancy calculated according to the Hayes–Nance analysis. Patients with trauma, congenital anomalies, craniofacial deformities, congenital tooth agenesis, severe facial asymmetry, patients for whom condylectomy was indicated, a history of previous orthodontic treatment, systemic diseases that may affect bone metabolism (such as diabetes mellitus), or the use of medications known to influence bone metabolism or orthodontic tooth movement (such as bisphosphonates, corticosteroids, or other systemic medications) were excluded from the study.

**Fig. 1:**
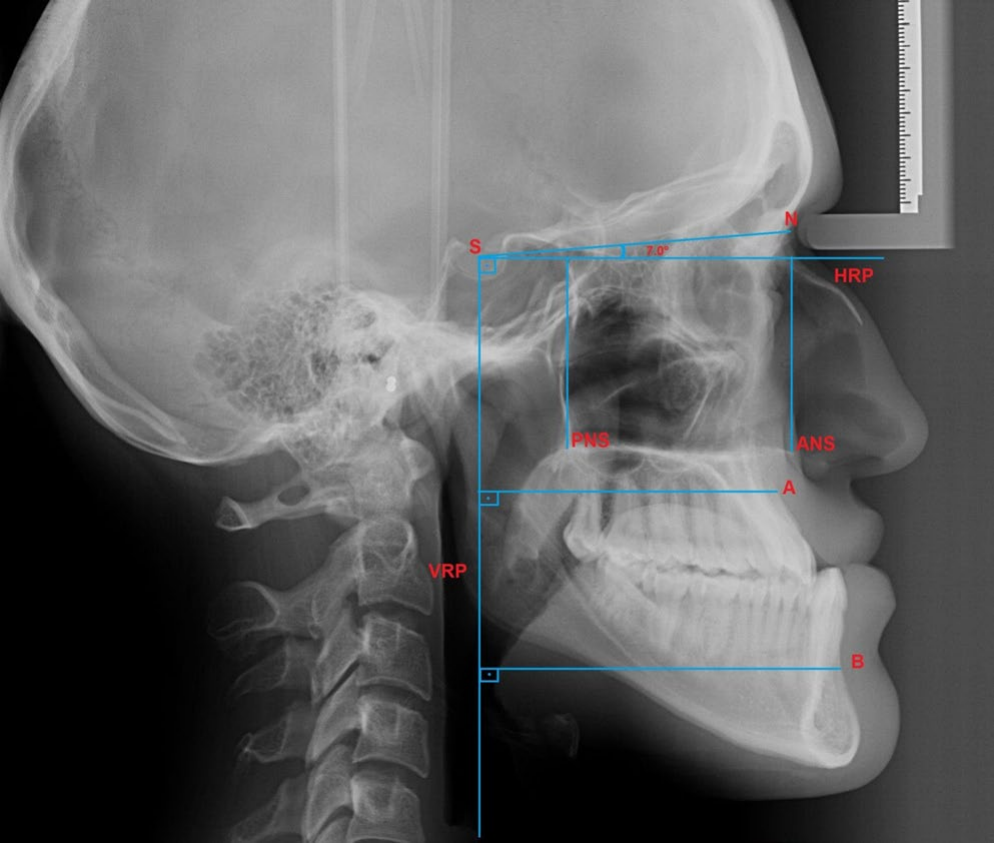
Reference planes and craniofacial landmarks used for the assessment of sagittal and vertical skeletal movements. *HRP* horizontal reference plane, *VRP* vertical reference plane, *S* sella, *N* nasion, *A* A point, *B* B point, *ANS* anterior nasal spine, *PNS* posterior nasal spine

The lateral cephalometric radiograph was obtained from each participant, and a horizontal reference plane (HRP) was established at a 7-degree angle to the sella-nasion horizontal plane. A vertical reference plane (VRP) passing through the sella and perpendicular to the HRP was determined. As shown in Fig. 1, the distances from the A and B points of the hard tissue to the VRP, and the distances from the anterior nasal spine (ANS) and posterior nasal spine (PNS) points to the HRP were evaluated. [19] The sagittal movement of the maxilla and mandible was determined by the difference in the vertical distance between the A and B points to the VRP, while the amount of impaction was determined by difference in the vertical distance of the ANS and PNS points to the HRP (Fig. 1) (Table 1). Baseline cephalometric parameters used for intergroup comparability, together with their definitions, are presented in Supplementary Table 1.

**Table 1:** A comparison of intergroup differences in the amount of surgical movement observed during treatment (T1-T0)

	SF		SE		*p* value
	Mean	SD	Mean	SD	
Max	3.64	1.50	3.47	2.77	0.849
Mand	−2.25	1.32	−1.20	1.75	0.112
ANS	−0.68	0.86	−1.43	1.42	0.128
PNS	−0.58	0.99	−0.63	1.30	0.930

An independent samples t-test was used for intergroup comparisons. *SF* surgery-first, *SE* surgery-early, *Max* maxilla, *Mand* mandible, *ANS* anterior nasal spine, *PNS* posterior nasal spine, *SD* standard deviation. Statistical significance was set at *p* < 0.05.

### SF approach

**Fig. 2:**
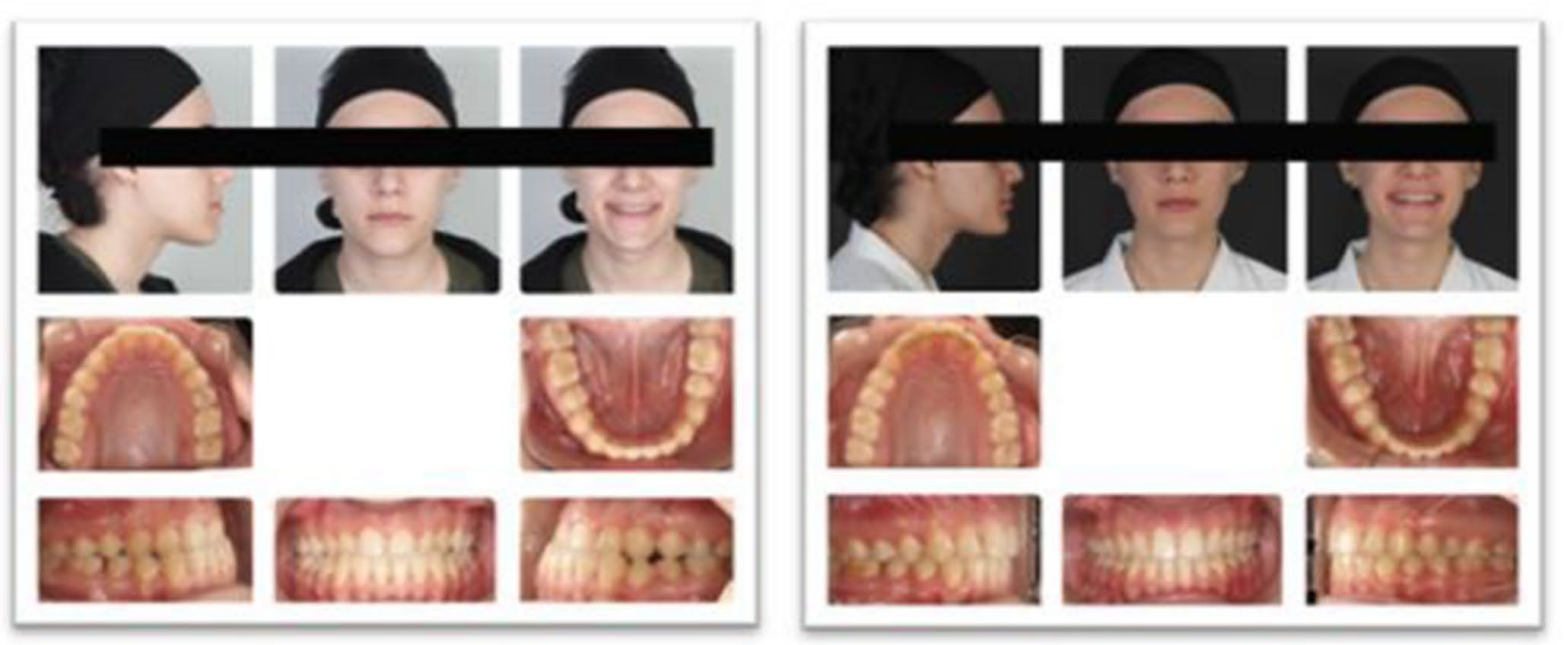
Pre- and post-treatment photographs of a patient treated with the surgery-first approach

No orthodontic preparation was performed before surgery (Fig. 2), and hard and soft tissue records were obtained at baseline (T0). Surgical splints were prepared using Nemo Studio software, and patients were referred for surgery. For intermaxillary fixation, eight mini-screws were used: four 1.6 mm in diameter and 8-mm in length Tomas^®^-pins SD self-drilling mini-screws (Dentaurum GmbH & Co. KG, Germany) were applied to the 4–5 and 2–3 regions of the upper and lower jaws. Bonding procedures for all patients were performed in the first month after surgery using American Orthodontics Master/Mini Master series brackets with a 0.018-inch slot and Roth prescription and Transbond XT (Light Cure Orthodontic Adhesive, 3M Unitek, Monrovia, Calif) for attachment. The sequence of NiTi archwires for both jaws was changed every 4–5 weeks, following the order of 0.014", 0.016", 0.016–0.016", and 0.016–0.022", and fixed orthodontic treatment was completed with a 0.016–0.022" SS archwire. At the end of treatment (T1), hard and soft tissue records were obtained at the debonding appointment when right-left Class I canine and molar relationships, as well as ideal overjet and overbite relationships, were achieved. The total treatment duration was 14.31 ± 1.16 months (Table 2).

**Table 2:** A comparison of treatment durations between the surgery-early and surgery-first groups

	SF		SE		*p* value
	Mean	SD	Mean	SD	
Treatment Duration (months)	14.31	1.16	15.23	1.17	0.068

An independent samples t-test was used for intergroup comparisons. Statistical significance was set at *p* < 0.05. *SF* surgery-first, *SE* surgery-early, *SD* standard deviation.

### SE approach

**Fig. 3:**
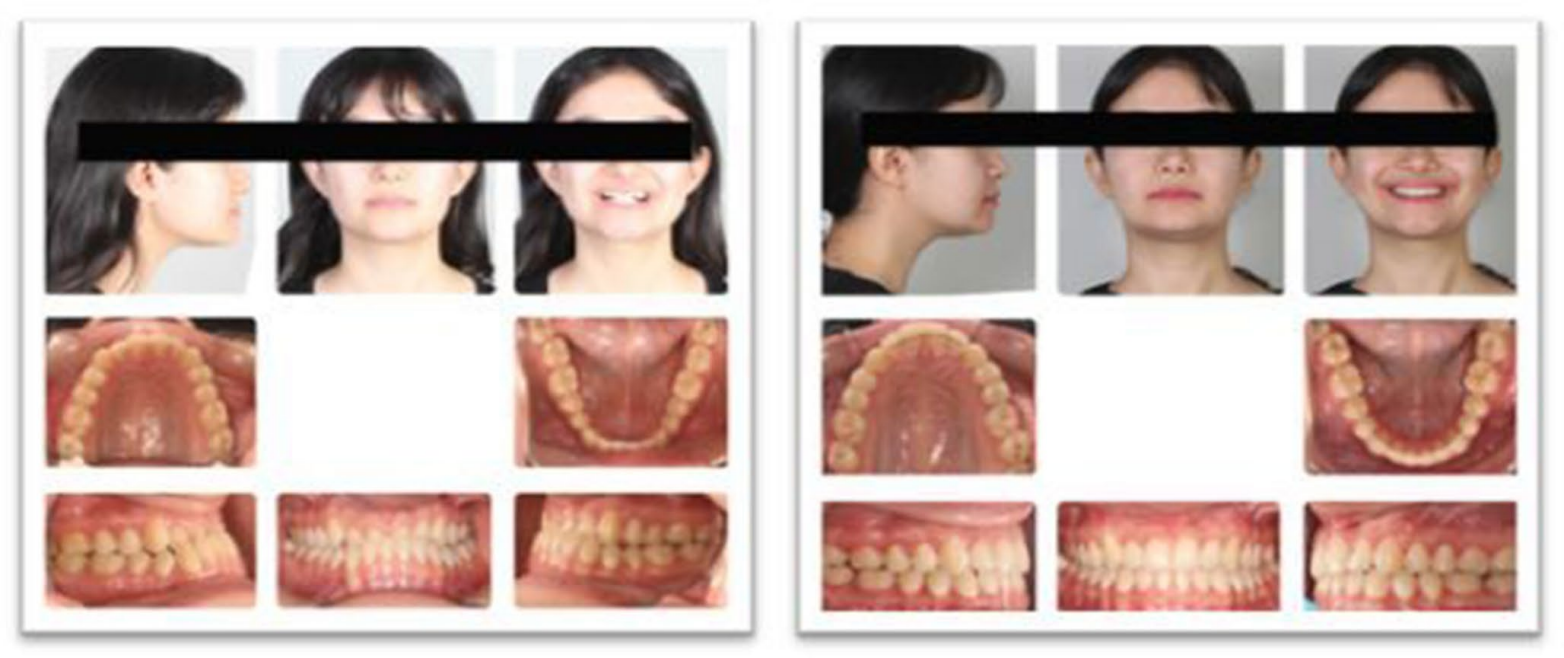
Pre- and post-treatment photographs of a patient treated with the surgery-early approach

At the first session, T0 records were obtained, and bonding for the upper and lower jaws was performed using American Orthodontics Master/Mini Master series brackets with a 0.018-inch slot and Roth prescription and Transbond XT (Light Cure Orthodontic Adhesive, 3M Unitek, Monrovia, Calif) (Fig. 3). The wire-changing sequence and intermaxillary fixation method were the same as in the SF group. The pre-surgical, fixed orthodontic treatment lasted 6.71 ± 0.76 months. Surgical splints were prepared using Nemo Studio software (version 2020, Nemotec, Madrid, Spain). For the surgical procedure, 0.016–0.022" SS archwires with crimpable hooks were used. These wires were prepared to allow intermaxillary fixation (IMF) elastics during surgery. Post-surgery, the surgical archwires were removed in the first month, and 0.016–0.022" NiTi wires were placed for settling. After 4–5 weeks, these were replaced with 0.016–0.022" SS wires. The post-surgical, fixed treatment lasted 8.51 ± 0.41 months, and the total treatment duration was 15.23 ± 1.17 months (Table 2). The T1 records were taken at the debonding appointment when right-left Class I canine and molar relationships and ideal overjet and overbite relationships were achieved.

### Elastic usage

In both groups, full-time elastic wear was recommended during post-surgical orthodontic treatment. Immediately after surgery, 3/16-inch, 3.5-oz posterior box elastics were used in the SF group via mini-screws, and in the SE group via surgical archwires. Full-time wear, including during meals, was maintained for one month, followed by another month of full-time use except during meals. [4]

### 3D recordings and measurements

To assess the effects of orthognathic surgery on dental and bony tissues, millimetric (length, dehiscence, and fenestration), volumetric measurements, and soft tissue anthropometric points were taken from routine pre-surgical (T0) and post-treatment (T1) cone beam computed tomography (CBCT) and 3dMD images.

### CBCT recordings

**Fig. 4:**
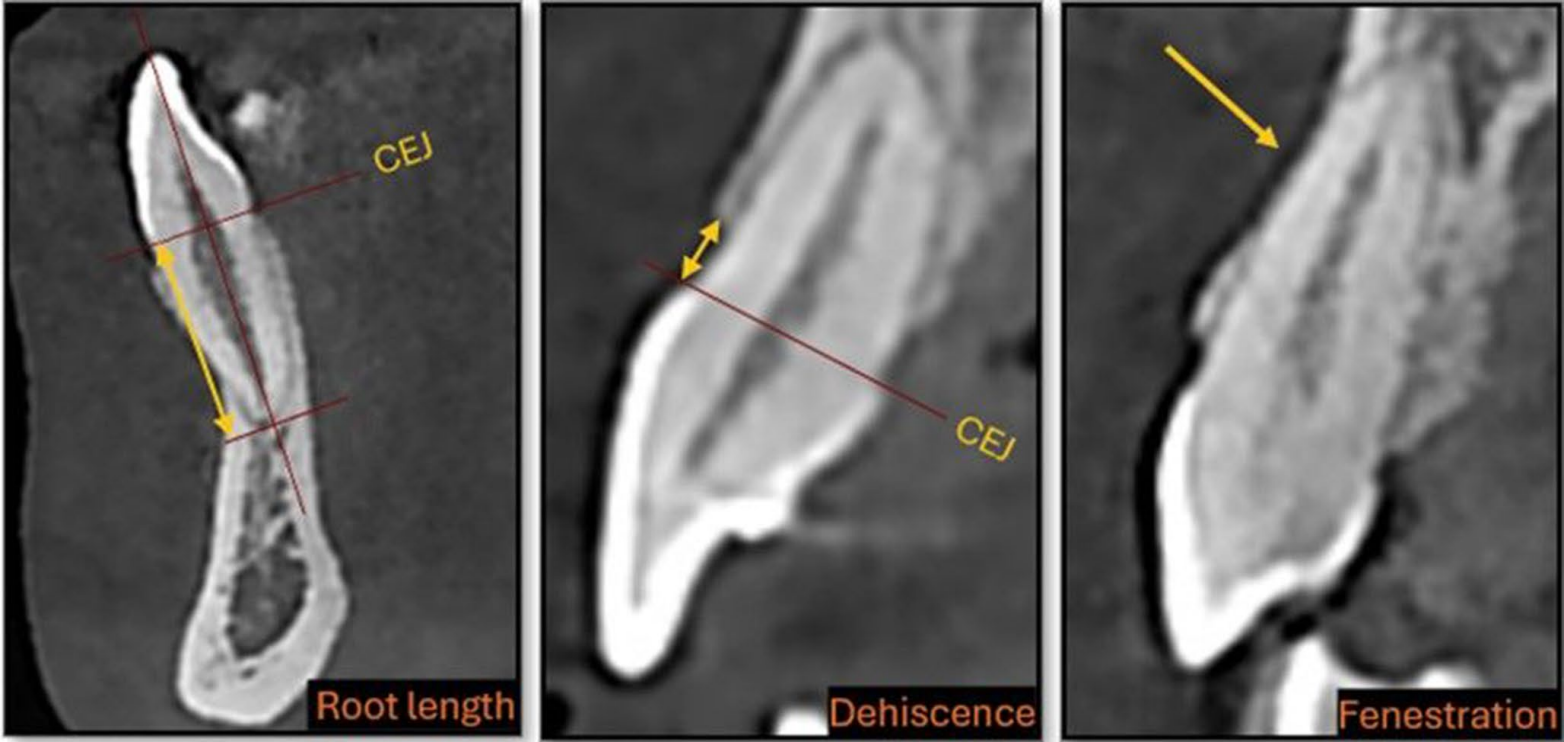
Assessment of root length and alveolar bone defects (dehiscence and fenestration) on CBCT images. *CEJ* cemento-enamel junction

**Fig. 5:**
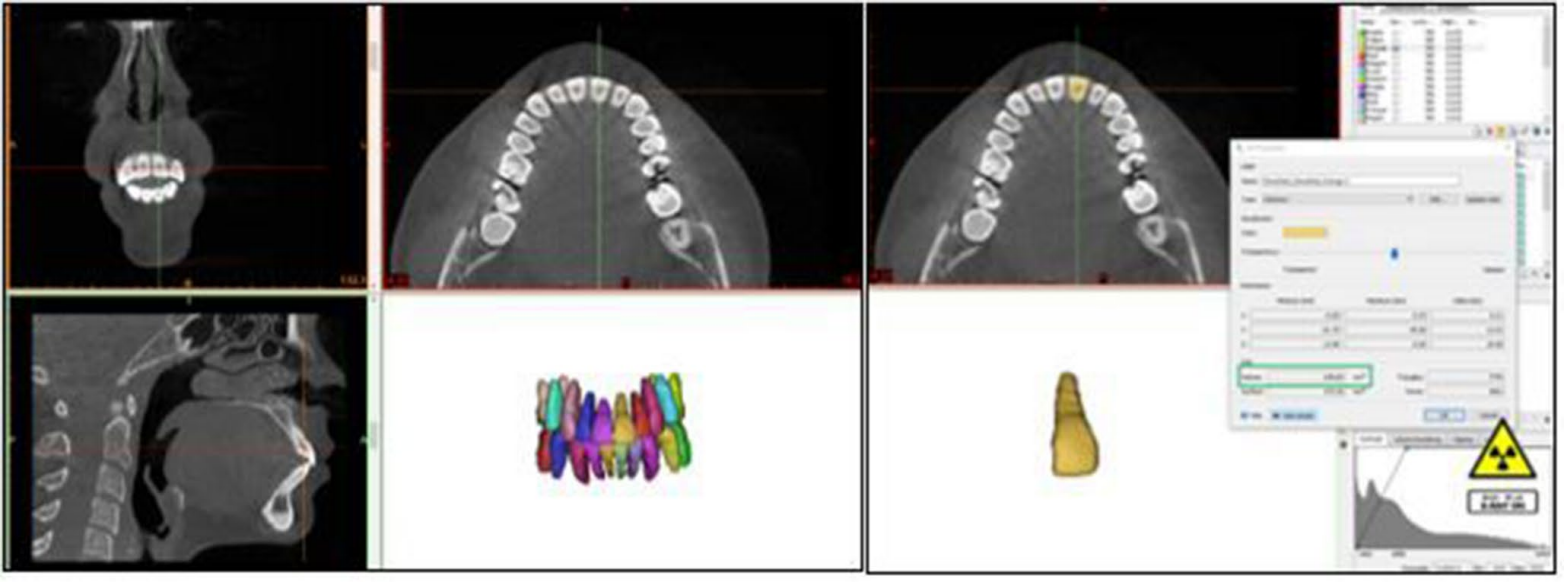
Three-dimensional segmentation and volumetric analysis of tooth roots using CBCT images

CBCT scans were obtained using NewTom 5G device (FP, Quantitative Radiology, Verona, Italy) with a voxel size of 0.25 mm³, a field of view of 12 × 8 cm, and an axial slice thickness of 0.25 mm. The scans were analyzed using NNT software to obtain coronal, sagittal, and axial sections, with the widest labiolingual section of the tooth chosen as the measurement plane. [20] Root length was calculated as the distance from the intersection of the long axis of the tooth and the line connecting the cementoenamel junction (CEJ) and root apex in the sagittal view (Fig. 4). [21] Alveolar bone height was classified as dehiscence if it exceeded 2 mm from the CEJ and as fenestration when the defect did not involve the alveolar crest. [22] The images were converted to DICOM format, and DICOM data were imported into the Mimics 18.0 (Materialise, Leuven, Belgium) program for volumetric measurements. To differentiate the teeth from surrounding tissues, the "Thresholding" value in the "Segment" section was set to Hounsfield Unit values between 1200–3071 for "Tooth" parameters, and a 3D model was generated after isolating the surrounding tissues. The same threshold range was applied for all segmentation procedures to ensure consistency across all measurements. The volume of the models was calculated using the "3D Properties" option and was repeated for all teeth (Fig. 5).

### 3D photographic recordings

**Fig. 6:**
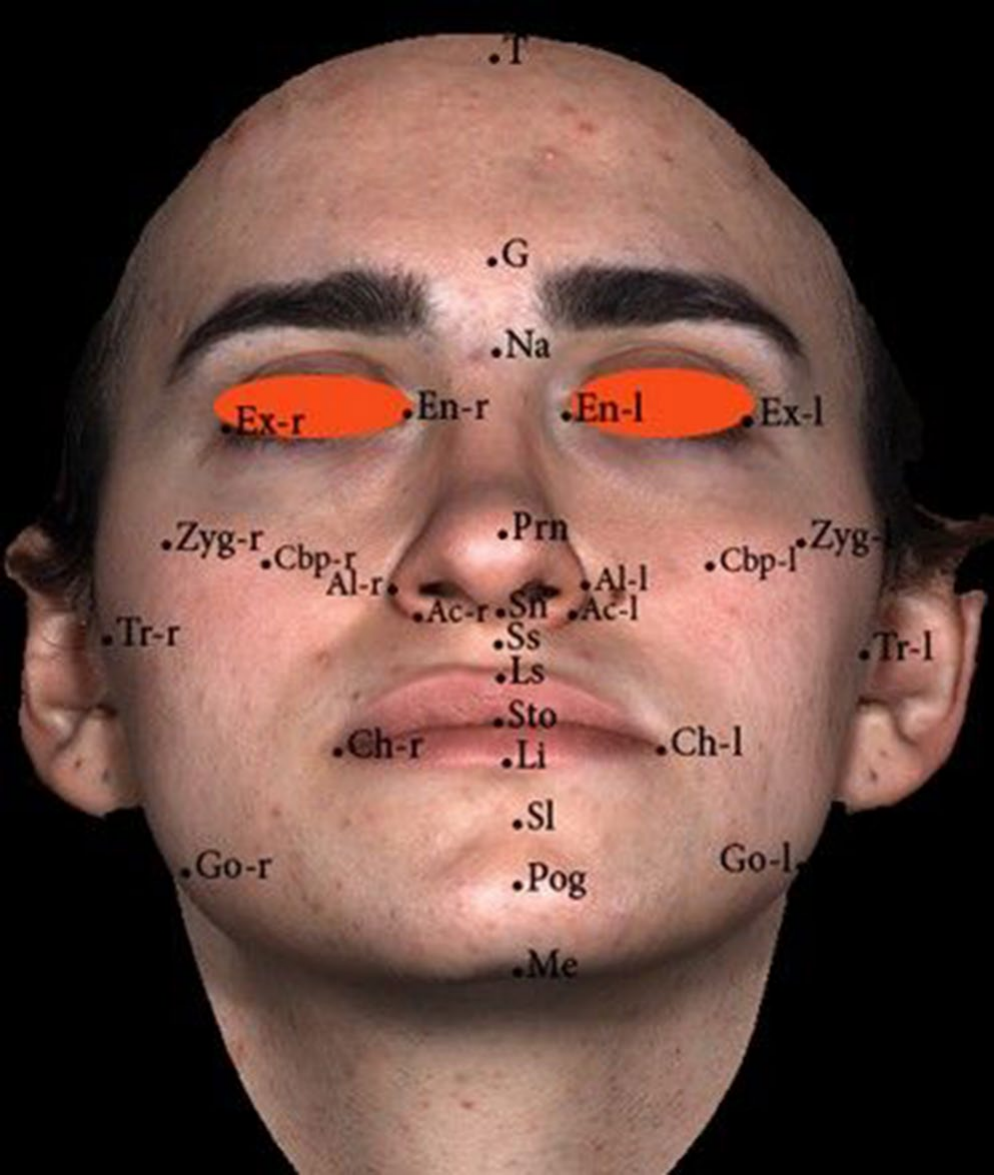
Anthropometric landmarks identified on three-dimensional (3dMD) stereophotogrammetric facial images for soft tissue analysis

Stereophotogrammetric images were obtained using 3dMD Face (3dMD™ Ltd, Atlanta, GA, USA) device. The 3dMD imaging system consists of two parts: three digital cameras and a flash. The images were captured while the patients were seated in an adjustable-height chair, with glasses and jewelry removed, and, for male patients, after shaving beards, if present. The chin was slightly tilted upwards from the natural head position during imaging. The images were saved in .tsb format, and measurements were made using the 3dMD Vultus (3dMD™ Ltd, Atlanta, GA, USA) software (Fig. 6). Blurry regions of the neck, ears, and hair were removed from the images. [23] Various anthropometric soft tissue changes in the vertical, sagittal, and transverse planes were recorded before and after orthognathic surgery. The processes of superimposition, landmarking, and acquisition of 3D data have been carried out as defined in the related study. [24] Landmark identification and subsequent manual corrections following superimposition were performed with knowledge of both the measurement timepoint and the treatment group.

### Method error assessment

All cephalometric, CBCT, and 3D stereophotogrammetric measurements in the present study were performed by the same researcher (HSK). Method error was assessed by repeating all CBCT measurements for 10 randomly selected patients after a 4-week washout period. The consistency of repeated assessments performed over time by the same researcher (HSK) was evaluated using intraclass correlation coefficient (ICC) method. Both linear and volumetric CBCT measurements were included in the intra-operator reliability analysis. The ICC values for CBCT measurements ranged from 0.929 to 1.000, indicating a strong positive correlation and high reliability, confirming that all measurements were reproducible.

### Statistical analysis

IBM SPSS Statistics software (version 21) was used for the analysis. The normality of data was first tested using the Shapiro-Wilk test. For within-group and between-group comparisons, two independent sample t-test or Mann-Whitney U test were used. A *p* value of < 0.05 was considered statistically significant.

## Results

No statistically significant differences were observed between the SF and SE groups in baseline cephalometric parameters (*p* > 0.05), indicating comparable skeletal and dentoalveolar characteristics at T0 (Supplementary Table 1).

### Hard and soft tissue measurements

The T0 measurements for hard tissue (tooth length, volume, and dehiscence) and soft tissue showed no significant sex-related differences within or between the groups (*p* > 0.05). Therefore, all data were combined and evaluated as a single group. Furthermore, when comparing T0 tooth length values between the two groups, no statistically significant difference was observed in the initial values (Table 3).

**Table 3:** Intergroup comparison of T0 tooth length measurements (mm)

Tooth	Root	SF		SE		*p* value
		Mean	SD	Mean	SD	
16	MB	12.46	1.54	11.63	1.04	0.139
16	DB	11.45	1.04	11.02	1.26	0.369
16	P	13.03	1.32	12.18	1.49	0.151
15		12.14	1.75	11.83	1.44	0.642
13		14.98	2.35	14.38	1.62	0.475
12		11.88	2.03	11.88	1.30	1.000
11		11.37	2.51	11.28	1.20	0.918
21		11.48	2.20	11.67	1.25	0.795
22		11.86	1.84	12.22	1.18	0.576
23		14.71	2.43	14.20	1.61	0.552
25		12.13	1.30	11.71	1.27	0.436
26	MB	12.31	1.38	11.36	0.89	0.058
26	DB	11.33	1.24	10.98	1.06	0.475
26	P	13.28	2.30	12.46	1.04	0.270
36	M	12.93	1.77	12.48	1.23	0.469
36	D	12.02	1.49	11.48	1.14	0.328
35		12.43	1.43	12.75	1.14	0.555
33		14.63	1.71	14.53	1.40	0.867
32		12.77	1.28	12.83	1.04	0.890
31		11.53	1.22	11.89	1.08	0.445
41		11.68	1.42	11.98	0.92	0.533
42		12.40	1.36	12.51	0.73	0.811
43		14.69	1.55	14.48	1.35	0.729
45		12.58	1.22	12.83	1.32	0.625
46	M	12.90	1.51	12.63	1.03	0.609
46	D	11.83	1.64	11.84	1.40	0.979

For intergroup comparisons, an independent samples t-test was used. For within-group comparisons, a paired-samples t-test was used. Statistical significance was set at *p* < 0.05. *SF* surgery-first, *SE* surgery-early, *SD* standard deviation, *MB* mesiobuccal, *DB* distobuccal, *P* palatal, *M* mesial, *D* distal.

Intra-group comparisons of soft tissue revealed that SF and SE demonstrated significant changes in the anteroposterior (z) plane at the pronasale (Prn), labiale superior (Ls), pogonion (Pog), and menton (Me) points, and in the horizontal (x) plane at the alare (Al) points. Following maxillary advancement surgery, the Prn and Ls points moved forward in the anteroposterior (z) plane, while the Al points exhibited changes in the horizontal (x) plane, expanding the nasal wings. After mandibular setback, Pog and Me points moved backward in the anteroposterior (z) direction. There were no statistically significant differences observed between the groups in the 3D movement of soft tissue landmarks at the end of treatment (Table 4).

**Table 4:** Intragroup and intergroup comparisons of the differences observed in soft tissue measurements (T1-T0)

AP	SF			SE			*p* value (intergroup)
	Mean	SD	*p* value	Mean	SD	*p* value	
G-z	−0.15	0.29	0.094	−0.01	0.22	0.880	0.189
N-z	−0.41	0.34	0.057	−0.08	0.29	0.356	0.160
En-r-z	0.15	0.64	0.436	−0.16	0.58	0.350	0.223
En-l-z	−0.08	0.44	0.552	−0.08	0.47	0.560	0.986
Prn-z	0.53	0.58	** 0.009**	1.13	0.93	**0.001**	0.052
Go-r-x	−0.93	0.81	0.082	−0.58	1.10	0.094	0.381
Go-r-y	0.33	2.27	0.625	−0.86	2.71	0.296	0.257
Go-r-z	−0.51	2.83	0.056	−0.57	2.68	0.473	0.942
Go-l-x	0.93	1.92	0.122	−0.35	1.44	0.423	0.079
Go-l-y	0.21	2.67	0.788	0.92	2.29	0.192	0.494
Go-l-z	−0.88	2.00	0.156	0.10	2.58	0.896	0.310
Sn-y	−0.16	1.24	0.661	0.29	1.47	0.513	0.428
Sn-z	0.21	2.83	0.801	1.32	2.84	0.135	0.347
Al-r-x	−1.43	1.72	** 0.015**	−0.90	0.99	**0.009**	0.371
Al-r-y	0.45	1.04	0.164	−0.05	1.49	0.917	0.357
Al-r-z	−1.51	4.60	0.281	1.30	4.68	0.356	0.153
Al-l-x	1.39	1.51	** 0.008**	2.01	2.80	**0.030**	0.509
Al-l-y	0.66	1.05	0.052	0.34	1.81	0.528	0.755
Al-l-z	−0.27	3.11	0.766	2.24	6.16	0.234	0.220
Ac-r-x	−0.15	1.19	0.678	0.04	1.50	0.935	0.266
Ac-r-y	0.45	0.83	0.089	0.66	1.46	0.146	0.843
Ac-r-z	0.85	1.80	0.129	0.70	4.40	0.593	0.590
Ac-l-x	0.18	0.60	0.325	0.62	1.85	0.267	0.437
Ac-l-y	0.63	1.23	0.089	0.63	1.54	0.187	0.443
Ac-l-z	0.98	2.27	0.061	0.87	3.02	0.341	0.514
Cc-z	0.21	0.74	0.340	0.92	1.56	0.067	0.172
Ss-y	−0.89	3.66	0.420	0.22	1.17	0.530	0.514
Ss-z	1.09	1.61	0.843	1.38	1.55	0.345	0.143
Cph-r-y	−0.41	1.97	0.488	−0.71	2.44	0.336	0.744
Cph-r-z	2.36	2.33	0.609	2.34	2.40	0.239	0.052
Cph-l-y	−0.21	1.94	0.717	−0.62	2.25	0.362	0.637
Cph-l-z	1.14	1.55	0.858	1.65	1.84	0.562	0.109
Ch-r-y	−0.35	2.14	0.582	0.24	2.08	0.694	0.499
Ch-r-z	−1.47	3.65	0.192	−0.43	3.20	0.648	0.470
Ch-l-y	0.08	1.75	0.871	0.00	2.10	0.994	0.911
Ch-l-z	−1.43	3.60	0.195	−1.11	2.97	0.222	0.811
Ls-y	1.20	1.97	0.492	0.93	1.61	0.243	0.696
Ls-z	2.14	1.77	** 0.002**	2.85	2.65	**0.003**	0.449
Li-y	1.40	3.40	0.182	−0.32	1.78	0.546	0.135
Li-z	−1.15	2.39	0.190	−1.42	2.79	0.105	0.118
Sto-y	−0.50	1.78	0.357	−0.36	1.78	0.499	0.855
Sto-z	−1.21	2.67	0.246	0.37	3.16	0.694	0.201
Sl-y	0.35	3.86	0.758	1.18	2.39	0.115	0.534
Sl-z	−1.08	2.39	0.196	−1.59	2.74	0.069	0.248
Pog-x	−0.29	2.06	0.638	−0.34	2.07	0.583	0.755
Pog-y	−0.53	2.02	0.386	−0.13	1.36	0.743	0.887
Pog-z	−3.06	2.68	** 0.002**	−1.89	2.01	**0.008**	0.291
Me-x	0.10	2.22	0.884	0.47	1.31	0.241	0.977
Me-y	0.74	1.52	0.333	0.47	1.34	0.279	0.382
Me-z	−4.23	2.95	**<0.001**	−3.17	2.45	**0.001**	0.348
Cbp-r-x	−0.11	0.30	0.232	−0.89	1.86	0.125	0.242
Cbp-r-y	0.06	0.24	0.409	0.29	1.70	0.563	0.977
Cbp-r-z	−0.02	0.47	0.905	−0.34	1.39	0.421	0.932
Cbp-l-x	−0.02	5.70	0.992	0.80	2.52	0.294	0.443
Cbp-l-y	0.23	0.56	0.190	−0.03	1.23	0.936	0.410
Cbp-l-z	−1.03	1.60	0.051	−0.20	1.03	0.519	0.078

A paired samples t-test was used for intragroup comparisons, and an independent samples t-test was used for intergroup comparisons. Statistical significance was set at p < 0.05. AP: anthropometric points; *SF* surgery-first, *SE* surgery-early, *SD* standard deviation, *r* right, *l* left.

Axis directions were defined as follows: x(horizontal)-axis left (no sign). right (minus sign); y (vertical)-axis. superior (no sign). inferior (minus sign); z(sagittal)-axis. anterior (no sign). posterior (minus sign) Abbreviations: *G* glabella, *N* nasion, *En* endocanthion, *Prn* pronasale, *Go* gonion, *Sn* subnasale, *Al* alare, *Ac* alar curvature point, *Cc* columella crest, *Ss* subspinale, *Cph* crista philtri, *Ch* cheilion, *Ls* labiale superius, *Li* labiale inferius, *Sto* stomion, *Sl* sublabiale, *Pg* soft tissue pogonion, *Me* menton, *Cbp* cheek bone point.

### Dehiscence and fenestration measurements

At the end of treatment, the root length and volume of all teeth significantly decreased in both groups (*p* < 0.05) (Tables 5 and 6), and the amount of dehiscence increased in all teeth with treatment (Table 7). These changes were more pronounced in the anterior teeth. After treatment, fenestration was observed in the SF group only in the maxilla at tooth 13, while in the SE group, fenestration was observed in the maxilla at teeth 13 and 15, and in the mandible, only at tooth 31. Overall, fenestration was infrequently observed, and no statistically significant differences were detected between the groups.

**Table 5:** Intragroup and intergroup comparisons of tooth length changes (T1–T0) (mm)

Tooth	Root	SF							SE							*p* value (intergroup)
		T0		T1		T1-T0			T0		T1		T1-T0			
		Mean	SD	Mean	SD	Mean	SD	*p* value	Mean	SD	Mean	SD	Mean	SD	*p* value	
16	**MB**	12.46	1.54	11.77	1.30	−0.69	0.68	** 0.005**	11.63	1.04	11.02	0.97	−0.62	0.46	**0.001**	0.754
16	**DB**	11.45	1.04	10.98	1.34	−0.47	0.89	0.096	11.02	1.26	10.59	1.23	−0.42	0.73	0.070	0.901
16	**P**	13.03	1.32	12.41	1.46	−0.63	0.66	** 0.007**	12.18	1.49	11.70	1.34	−0.47	0.70	**0.038**	0.593
15		12.14	1.75	11.38	1.54	−0.76	0.55	** 0.001**	11.83	1.44	11.53	1.20	−0.31	0.54	0.071	0.054
13		14.98	2.35	13.90	2.38	−1.08	0.69	**<0.001**	14.38	1.62	13.45	1.76	−0.92	0.76	**0.001**	0.619
12		11.88	2.03	11.12	1.88	−0.76	0.66	** 0.002**	11.88	1.30	11.29	1.44	−0.58	0.44	**0.001**	0.456
11		11.37	2.51	10.34	1.99	−1.03	1.21	** 0.013**	11.28	1.20	10.63	1.33	−0.65	0.55	**0.002**	0.337
21		11.48	2.20	10.51	2.29	−0.97	0.84	** 0.002**	11.67	1.25	10.93	1.22	−0.73	0.67	**0.003**	0.459
22		11.86	1.84	10.90	1.44	−0.96	0.99	** 0.006**	12.22	1.18	10.98	1.58	−1.24	0.98	**0.001**	0.488
23		14.71	2.43	13.92	2.42	−0.79	0.41	**<0.001**	14.20	1.61	13.63	1.62	−0.57	0.55	**0.004**	0.268
25		12.13	1.30	11.60	1.58	−0.53	0.54	** 0.006**	11.71	1.27	11.35	1.37	−0.36	0.36	**0.005**	0.380
26	**MB**	12.31	1.38	11.54	1.43	−0.77	0.75	** 0.005**	11.36	0.89	10.87	0.99	−0.49	0.46	**0.003**	0.290
26	**DB**	11.33	1.24	10.60	1.19	−0.73	0.69	** 0.004**	10.98	1.06	10.47	1.05	−0.52	0.54	**0.007**	0.416
26	**P**	13.28	2.30	12.33	1.73	−0.95	1.18	** 0.018**	12.46	1.04	12.00	1.09	−0.46	0.61	**0.025**	0.215
36	**M**	12.93	1.77	12.23	1.47	−0.71	0.71	** 0.005**	12.48	1.23	11.81	0.95	−0.67	0.65	**0.005**	0.882
36	**D**	12.02	1.49	11.42	1.37	−0.60	0.55	** 0.003**	11.48	1.14	10.85	1.14	−0.63	0.80	**0.021**	0.930
35		12.43	1.43	11.59	1.77	−0.84	0.84	** 0.005**	12.75	1.14	12.47	1.29	−0.28	0.26	**0.003**	**0.039**
33		14.63	1.71	13.95	1.66	−0.68	0.56	** 0.001**	14.53	1.40	13.78	1.59	−0.75	0.56	**0.001**	0.773
32		12.77	1.28	11.97	1.39	−0.80	0.56	**<0.001**	12.83	1.04	12.18	0.95	−0.65	0.65	**0.005**	0.500
31		11.53	1.22	10.43	1.19	−1.09	0.54	**<0.001**	11.89	1.08	11.38	1.13	−0.52	0.42	**0.001**	**0.008**
41		11.68	1.42	11.02	1.49	−0.66	0.54	** 0.001**	11.98	0.92	11.33	1.09	−0.65	0.51	**0.001**	0.969
42		12.40	1.36	11.68	1.43	−0.73	0.44	**<0.001**	12.51	0.73	11.69	1.12	−0.82	0.69	**0.002**	0.700
43		14.69	1.55	13.80	1.87	−0.89	0.66	** 0.001**	14.48	1.35	13.54	1.58	−0.94	0.83	**0.002**	0.872
45		12.58	1.22	12.16	1.46	−0.42	0.39	** 0.004**	12.83	1.32	12.20	1.52	−0.63	0.63	**0.005**	0.321
46	**M**	12.90	1.51	12.51	1.52	−0.39	0.31	** 0.001**	12.63	1.03	11.87	0.93	−0.76	0.64	**0.002**	0.088
46	**D**	11.83	1.64	11.44	1.69	−0.38	0.38	** 0.005**	11.84	1.40	11.48	1.34	−0.36	0.40	**0.011**	0.878

A paired-samples t-test was used for intragroup comparisons. An independent samples t-test was used for intergroup comparisons. Statistical significance was set at *p* < 0.05. *SF* surgery-firs, *SE* surgery-early, *SD* standard deviation, *MB* mesiobuccal, *DB* distobuccal, *P* palatal, *M* mesial, *D* distal

**Table 6:** Intragroup and intergroup comparisons of tooth volume changes after treatment (T1–T0) (mm³)

Tooth	SF							SE							*p* value (intergroup)
	T0		T1		T1-T0			T0		T1		T1-T0			
	Mean	SD	Mean	SD	Mean	SD	***p*** value	Mean	SD	Mean	SD	Mean	SD	***p*** value	
11	701.26	128.47	666.62	114.39	−34.65	27.11	**0.002**	625.23	126.81	600.43	109.51	−24.80	31.80	**0.002**	0.219
12	529.02	145.80	504.00	134.32	−25.01	23.49	**0.002**	444.49	107.81	426.93	100.87	−17.57	21.01	**0.002**	0.347
13	764.55	188.15	741.23	188.25	−23.32	36.88	**0.002**	646.64	155.76	632.67	160.81	−13.97	12.22	**0.002**	0.443
15	648.16	104.28	622.99	96.54	−25.16	27.14	**0.002**	551.03	120.61	536.87	114.42	−14.16	14.47	**0.002**	0.843
16	1354.68	207.77	1285.27	194.82	−69.41	92.97	**0.015**	1138.93	194.36	1097.50	171.89	−41.43	46.64	**0.002**	0.478
21	693.45	123.55	676.92	122.47	−16.53	12.38	**0.002**	627.20	122.78	608.55	116.44	−18.65	18.24	**0.002**	1.000
22	452.09	121.26	440.44	118.63	−11.65	8.17	**0.002**	447.29	113.55	424.67	116.94	−22.62	8.81	**0.002**	**0.003**
23	719.55	233.02	695.83	240.74	−23.73	26.10	**0.002**	645.38	141.82	616.10	156.28	−29.28	22.12	**0.002**	0.291
25	656.56	131.27	638.22	124.26	−18.34	12.88	**0.002**	570.04	110.19	541.90	109.69	−28.14	36.68	**0.002**	0.671
26	1343.37	248.88	1255.84	199.47	−87.52	82.93	**0.002**	1148.80	201.11	1106.51	198.37	−42.28	56.43	**0.002**	0.068
31	352.64	62.77	337.82	47.00	−14.82	22.96	**0.002**	319.76	62.08	307.44	58.23	−12.33	14.71	**0.002**	0.932
32	403.47	73.40	370.49	62.31	−32.98	23.89	**0.002**	383.43	79.48	358.06	73.92	−25.37	27.01	**0.002**	0.478
33	674.64	182.74	635.51	163.06	−39.14	34.04	**0.002**	608.45	155.46	567.12	160.38	−41.33	53.44	**0.002**	0.671
35	636.82	114.37	606.59	101.96	−30.23	29.50	**0.002**	537.05	105.45	521.62	101.85	−15.43	16.89	**0.002**	0.114
36	1303.81	251.12	1225.62	205.32	−78.18	88.00	**0.002**	1161.57	201.13	1118.20	211.96	−43.38	56.39	**0.002**	0.378
41	363.49	63.52	336.11	54.70	−27.38	27.99	**0.002**	353.97	102.50	341.46	101.48	−12.51	12.23	**0.002**	0.219
42	401.94	68.49	389.12	57.95	−12.83	19.46	**0.023**	390.07	79.23	361.18	60.38	−28.89	40.90	**0.002**	0.219
43	656.58	189.87	630.12	184.19	−26.46	34.71	**0.002**	577.09	142.18	562.38	137.11	−14.71	20.28	**0.002**	0.671
45	637.48	119.21	601.40	92.19	−36.08	38.21	**0.002**	555.68	94.68	533.89	103.88	−21.78	28.89	**0.002**	0.551
46	1289.92	270.04	1236.81	209.49	−53.11	96.58	**0.028**	1116.49	188.18	1073.23	187.77	−43.26	47.95	**0.003**	0.319

A Wilcoxon signed-rank test was used for intragroup comparisons. A Mann–Whitney U test was used for intergroup comparisons. Statistical significance was set at *p* < 0.05. *SF* surgery-first, *SE* surgery-early, *SD* standard deviation

**Table 7:**
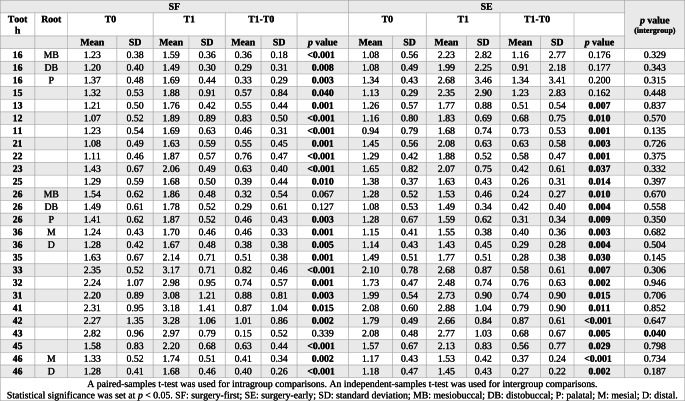
Intragroup and intergroup comparisons of changes in dehiscence (T1–T0) (mm)

In summary, both treatment approaches were associated with reductions in root length and volume and with increased dehiscence, particularly in anterior teeth, whereas fenestration was infrequently observed. Soft tissue landmarks demonstrated predictable positional changes following maxillary advancement and mandibular setback. However, no statistically significant differences were identified between the SF and SE groups in either hard or soft tissue outcomes.

## Discussion

This prospective, randomized study aimed to investigate the effects of surgical timing on root volume, root length, dehiscence, fenestration, and soft tissues in similar bimaxillary Class III patient groups using 3D analysis. As no significant differences were observed between the groups (with few exceptions), the present study failed to reject the null hypothesis.

The absence of significant differences in baseline cephalometric measurements, particularly those reflecting incisor position and inclination, sagittal skeletal relationships, and overjet, indicates that the SF and SE groups were well matched at baseline. The groups were also comparable in terms of age, treatment duration, and the magnitude of surgical movements, which minimized potential confounding effects in the subsequent comparisons.

Apical root resorption is influenced by various factors such as genetic variables, trauma, age, sex, nutrition, orthodontic treatment method, the magnitude of applied forces, treatment duration, and tooth length and morphology. [25] Apical root resorption has also been observed in 7% to 15% of individuals who have not undergone any orthodontic intervention. [9] The orthodontic mechanics used, intraoral elastics, or trauma from the failure to achieve postoperative ideal occlusion also play a role in root resorption. [26] Furthermore, postoperative blood flow reduction can lead to necrosis of bone and soft tissues, tooth devitalization, and periodontal loss; however, because vascular problems are generally temporary in nature, serious side effects are less likely to occur. [6] It should be emphasized that root resorption resulting from orthodontic treatment, when less than 1 mm, appeared to have limited impact on the functional and biomechanical integrity of the teeth in the present study. Although statistically significant reductions in root length and volume were observed, the magnitude of these changes was relatively small. In the present study, root resorption was generally less than 1 mm (ranging from − 1.24 to − 0.28 mm), which is considered unlikely to compromise the functional and biomechanical integrity of the teeth. Therefore, while some findings reached statistical significance, the observed changes should be interpreted as clinically minor rather than clinically critical. Root resorption was observed in all teeth in both groups; however, a loss of root length exceeding 1 mm was only detected in four teeth, all of which were anterior. Reports from the literature have suggested that the force applied to the incisors during fixed orthodontic treatment has a more direct effect, and, therefore, resorption activity is frequently observed in these teeth. [25, 27] Recent CBCT studies have also supported this finding. [17, 21] Studies in the literature have also indicated that there is no difference in root resorption between the sexes [25], and, in the present study, no statistically significant difference between males and females was found in either group (*p* > 0.05). While tooth movement was expected to be completed more quickly in the SF group due to the increase in the metabolic cycle after surgery (the RAP phenomenon), the duration of orthodontic treatment was similar to the SE group (an average 15 months). The reasons for this may be as follows: first, the RAP phenomenon is valid for a limited period of three to four months after surgery [11], which is too short to meet completion conditions. Second, tooth movement includes not only the pressure and tension regions under the influence of orthodontic mechanics and devices but the physiological and transformational changes in the surrounding bone architecture as well. [12]

Flavio et al. recommended the use of elastics for at least two months post-surgery in patients treated with an SF approach to prevent relapse. [28] According to Proffit, elastic use after surgery is important for maintaining occlusal stability and providing stabilization. Elastics that apply light force are preferred, and excessive force should be avoided, as this can jeopardize surgical stability and negatively affect healing. Proffit et al. also suggested an approach based on clinical observation rather than fixed durations. In the initial postoperative visit, medium elastics were provided in vertical and box configurations with continuous use—including during meals. [4] The literature has explained the association between long-term use of intermaxillary elastics, prolonged treatment duration, and retraction mechanics with root resorption. However, since the relevant protocols were not used in the present study, the observed root resorption remained at approximately 1 mm [29].

Strippoli et al., in a study of 12 adult patients, combined orthodontics with piezo-incisions and evaluated apical root resorption, alveolar bone height, and thickness using CBCT. [21] They noted significantly more severe resorption in the anterior teeth compared to the posterior ones in the maxilla and mandible. Castro et al. assessed root resorption using CBCT in 30 patients with Class I malocclusion who underwent non-extraction orthodontic treatment and found that 46% of the treated roots exhibited root resorption. The greatest resorption occurred in the incisors and the distal roots of the first molars in both the upper and lower dentition. However, they also pointed out that factors such as the direction of tooth movement, type of applied force, and its magnitude could influence resorption. [30] In the present study, resorption in the incisors was slightly higher than in other teeth, while the first molars showed lower levels of resorption. This could be due to limited tooth movement in the posterior region as no extractions were performed in the study groups.

A study using CBCT images before and 6–12 months after orthognathic surgery in patients with skeletal Class II and III malocclusions reported statistically significant reductions in root length and volume within groups, but no differences between groups, indicating that these changes were independent of the type of osteotomy and the anomaly type. [5] The results of the present study appear to be in line with this report, as orthognathic surgery was associated with statistically significant, but clinically minor, reductions in tooth root length and volume.

Dehiscence refers to bone defects extending to the cervical margin of the alveolar crest, while fenestration refers to localized, round, window-like bone defects that do not extend to the alveolar crest. [31] These defects, which are risk factors for gingival recession characterized by exposure of the root surface in the cervical region, can lead to aesthetic and instability problems ranging from discoloration and dentin sensitivity to root caries and resorption. They can be influenced by the direction of tooth movement, the characteristics of orthodontic forces, a thin alveolar bone structure, sex, age, crowding, and skeletal patterns. [22] In the present study, attention was given to the close similarities between these influencing factors, and the superiority of CBCT over two-dimensional imaging methods for evaluating these bone defects (with high precision) was highlighted. [32] Dos Santos et al. evaluated dehiscence prevalence in Class II and III patients undergoing bimaxillary orthognathic surgery using CBCT and reported a slight increase (5%) post-surgery. [33] The researchers attributed the postoperative increase to temporary vascularization loss, the traumatic effects of surgery on tissues, and difficulties in maintaining oral hygiene. [33] Yağcı et al. examined dehiscence and fenestration prevalence in patients with different malocclusions and noted that fenestration was more common in the maxilla, while dehiscence showed higher prevalence in the mandible. [22, 34] In the present study, while a significant increase in dehiscence was observed in all teeth, no increase greater than 2 mm was detected, and fenestration was rare (one tooth in the SF group and three in the SE group). The low prevalence of alveolar bone defects may be attributed to the non-extraction cases, absence of expansion, the lack of mini-screws used to support dental movements, absence of active periodontal disease in the patients, and the relatively short treatment duration.

The traditional view of the biological basis of orthodontic tooth movement is that alveolar bone follows the tooth movement. However, recent studies have shown that the alveolar bone does not simply follow tooth movement but, rather, that orthodontic tooth movement leads to alveolar bone loss. [35] In one study, it was reported that alveolar bone height and root lengths continued to decrease after surgery, although significant alveolar bone loss primarily occurred during the pre-surgical orthodontic phase with intensive tooth movements. Additionally, it has been reported that alveolar bone turnover slows down with tooth movement, affecting bone resorption and formation. [31] Within the limitations of the present study, no clear superiority was observed between performing orthodontic treatment before or after surgery in terms of these parameters.

Baik et al. evaluated soft tissue changes in Class III surgical patients post-surgery and reported that the Prn and Ls points moved forward, while the Pog and Me points moved backward. [19] Çoban et al., [24] examined the 3D soft tissue changes in patients undergoing bimaxillary orthognathic surgery. Although the amount of surgical movement differed, the direction of movement showed similarities with the findings of the present study. It should be kept in mind that changes in soft tissues following orthognathic surgery can vary based on various factors, including the individual’s anatomical structure, age, sex, and personal characteristics.

### Limitations and clinical recommendations

A non-surgical control group was not included because it would be ethically inappropriate to withhold or delay indicated surgical treatment in patients with significant skeletal Class III discrepancies. Furthermore, acquiring CBCT and lateral cephalometric radiographs solely for control purposes would entail unnecessary radiation exposure and would not be justified. Therefore, the findings should be interpreted within the context of surgically treated patients only. The limitations of the present study also include the relatively small sample size (*n* = 24) and the lack of a long-term follow-up, which limits the assessment of long-term stability and progression of root resorption. In addition, although no statistically significant baseline differences were detected, the unequal gender distribution between the SF and SE groups should be considered when interpreting the results. Furthermore, the clinical trial was registered retrospectively because the formal trial registration was completed after the study had commenced. This may be considered a limitation in terms of research protocol rigour. However, ethical approval had been obtained prior to patient recruitment, and the study protocol, eligibility criteria, and outcome measures had been predefined before the initiation of the study. Another limitation of the present study is the absence of quantitative periodontal indices (e.g., plaque index or bleeding on probing), which could be addressed in future studies. Because landmark identification and manual corrections following superimposition were conducted without blinding to the measurement timepoint or treatment allocation, observer-related bias cannot be completely ruled out. However, the reliability analysis demonstrated excellent reproducibility of the measurements.

Although some findings reached statistical significance, the magnitude of most observed root resorption and alveolar bone changes was limited and unlikely to be clinically critical. From a clinical standpoint, such minor changes are frequently encountered during orthodontic–surgical treatment and are not expected to adversely affect tooth prognosis or overall treatment outcomes when appropriate planning and monitoring are implemented.

The findings of the present study are most applicable to similar, well-selected skeletal Class III patients treated under comparable clinical conditions. Larger prospective studies are still needed to confirm these findings.

## Conclusions

Considering the limitations of the present study, the following conclusions can be drawn:

1. In patients with skeletal Class III malocclusion, reductions in root volume and length were observed following bimaxillary orthognathic surgery. The timing of the surgery did not appear to substantially affect these changes.
2. Both groups showed more dehiscence in the anterior teeth compared to the posterior ones after surgery. No significant differences were observed between the groups in terms of dehiscence and fenestration.
3. The 3D changes in soft tissue anthropometric points were as follows: the Al base (bilateral Ac) widened, the nose tip (Prn) and upper lip (Ls) moved forward, while the chin tip (Pog) and Me moved backward. These 3D changes were observed regardless of surgical timing within the scope of the present study.
4. No significant differences were observed in the effectiveness of surgery performed before or after orthodontic treatment in terms of root length, alveolar bone, or soft tissue changes.

When surgery-first and surgery-early approaches were compared, no significant differences were observed in hard or soft tissue changes. For optimal surgical timing, patient selection with appropriate indications, meticulous treatment planning, and effective communication between the surgeon and orthodontist are critical.

## Supplementary Information

Below is the link to the electronic supplementary material.

Supplementary Material 1 (DOCX 16.1 KB)

## Data Availability

No datasets were generated or analysed during the current study.

## References

[1] Soncul M, Bamber MA (2004) Evaluation of facial soft tissue changes with optical surface scan after surgical correction of class III deformities. J Oral Maxillofac Surg 62:1331–1340. 10.1016/j.joms.2004.04.019 15510353 10.1016/j.joms.2004.04.019

[2] Van Loon B, Van Heerbeek N, Bierenbroodspot F et al (2015) Three-dimensional changes in nose and upper lip volume after orthognathic surgery. Int J Oral Maxillofac Surg 44:83–9. 10.1016/j.ijom.2014.08.001 25218802 10.1016/j.ijom.2014.08.001

[3] Juggins KJ, Nixon F, Cunningham SJ (2005) Patient-and clinician-perceived need for orthognathic surgery. Am J Orthod Dentofacial Orthop 128:697–702. 10.1016/j.ajodo.2004.09.022 16360908 10.1016/j.ajodo.2004.09.022

[4] Proffit WR, White RP, Sarver DM (2003) Contemporary treatment of dentofacial deformity. Mosby, St. Louis, p 24. Chap. 1

[5] Erdem ME, Irgın C (2025) Does orthognathic surgery affect tooth root length and volume: a retrospective cohort study. Angle Orthod 95:188–198. 10.2319/052024-390.1 39799969 10.2319/052024-390.1 PMC11842105

[6] Patel PK, Morris DE, Gassman A (2007) Complications of orthognathic surgery. J Craniofac Surg 18:975–85. 10.1097/scs.0b013e318068442c 17667699 10.1097/scs.0b013e318068442c

[7] Gaszyńska E, Kozakiewicz M (2008) Complications of surgical treatment of mandibular prognathism. Pol Merkur Lekarski 25:27–31 18839610

[8] Emshoff R, Kranewitter R, Gerhard S, Norer B, Hell B (2000) Effect of segmental Le fort I osteotomy on maxillary tooth type-related pulpal blood-flow characteristics. Oral Surg Oral Med Oral Pathol Oral Radiol 89:749–752. 10.1067/moe.2000.106691 10.1067/moe.2000.106691 10846132

[9] Çoban G, Gül Amuk N, Yağcı A, Akgün G, Abbood Abbood IH (2023) Evaluation of external apical root resorption caused by fixed functional treatment of class II malocclusion: cast splint Herbst appliance vs. Forsus fatigue resistant device. J Orofac Orthop 84:50–59. 10.1007/s00056-021-00334-x 34331069 10.1007/s00056-021-00334-x

[10] Lee R (1994) The benefits of post-surgical orthodontic treatment. Br J Orthod 21:265–274. 10.1179/bjo.21.3.265 7947581 10.1179/bjo.21.3.265

[11] Liou EJ, Chen PH, Wang YC, Yu CC, Huang C, Chen YR (2011) Surgery-first accelerated orthognathic surgery: postoperative rapid orthodontic tooth movement. J Oral Maxillofac Surg 69:781–785. 10.1016/j.joms.2010.10.035 21353934 10.1016/j.joms.2010.10.035

[12] Hernández-Alfaro F, Guijarro-Martínez R, Peiró-Guijarro MA (2014) Surgery first in orthognathic surgery: what have we learned? A comprehensive workflow based on 45 consecutive cases. J Oral Maxillofac Surg 72:376–390. 10.1016/j.joms.2013.08.013 24139292 10.1016/j.joms.2013.08.013

[13] Hernández-Alfaro F, Guijarro-Martínez R (2014) On a definition of the appropriate timing for surgical intervention in orthognathic surgery. Int J Oral Maxillofac Surg 43:846–55. 10.1016/j.ijom.2014.02.007 24631424 10.1016/j.ijom.2014.02.007

[14] Naini FB, Gill DS (2017) Orthognathic surgery: principles, planning and practice. John Wiley & Sons, Chichester, Chap 42:670

[15] Millesi GA, Zimmermann M, Eltz M (2023) Surgery first and surgery early treatment approach in orthognathic surgery. Oral Maxillofac Surg Clin North Am 35:71–82. 10.1016/j.coms.2022.06.010 36336597 10.1016/j.coms.2022.06.010

[16] Huang C, Hsu S, Chen YR (2014) Systematic review of the surgery-first approach in orthognathic surgery. Biomed J 37:184–190. 10.4103/2319-4170.126863 25116713 10.4103/2319-4170.126863

[17] Chen J, Ning R (2023) Evaluation of root resorption in the lower incisors after orthodontic treatment of skeletal Class III malocclusion by three-dimensional volumetric measurement with cone-beam computed tomography. Angle Orthod 93:320–327. 10.2319/090322-609.1 36780279 10.2319/090322-609.1 PMC10117204

[18] Atilla AO, Ozturk T, Eruz MM, Yagci A (2020) A comparative assessment of orthodontic treatment outcomes using the quantitative light-induced fluorescence (QLF) method between direct bonding and indirect bonding techniques in adolescents: a single-centre, single-blind randomized controlled trial. Eur J Orthod 42:441–453. 10.1093/ejo/cjz058 31375814 10.1093/ejo/cjz058

[19] Baik H-S, Kim SY (2010) Facial soft-tissue changes in skeletal class III orthognathic surgery patients analyzed with 3-dimensional laser scanning. Am J Orthod Dentofacial Orthop 138:167–178. 10.1016/j.ajodo.2010.02.022 20691358 10.1016/j.ajodo.2010.02.022

[20] Sun L, Mu C, Chen L, Zhao B, Pan J, Liu Y (2022) Dehiscence and fenestration of class I individuals with normality patterns in the anterior region: a CBCT study. Clin Oral Invest 26:4137–4145. 10.1007/s00784-022-04384-2 10.1007/s00784-022-04384-2 PMC9072473 35254527

[21] Strippoli J, Schmittbuhl M, Durand R et al (2021) Impact of piezocision-assisted orthodontics on root resorption and alveolar bone: a prospective observational study. Clin Oral Invest 25:4341–8. 10.1007/s00784-020-03282-9 10.1007/s00784-020-03282-9 34037852

[22] Yagci A, Veli İ, Uysal T, Ucar FI, Ozer T, Enhos S (2012) Dehiscence and fenestration in skeletal class I, II, and III malocclusions assessed with cone-beam computed tomography. Angle Orthod 82:67–74. 10.2319/040811-250.1 21696298 10.2319/040811-250.1 PMC8881026

[23] Baysal A, Ozturk MA, Sahan AO, Uysal T (2016) Facial soft-tissue changes after rapid maxillary expansion analyzed with 3-dimensional stereophotogrammetry: a randomized, controlled clinical trial. Angle Orthod 86:934–942. 10.2319/111315-766.1 27058647 10.2319/111315-766.1 PMC8597340

[24] Çoban G, Yavuz İ, Demirbaş AE (2021) Three-dimensional changes in the location of soft tissue landmarks following bimaxillary orthognathic surgery. J Orofac Orthop 82:257–265. 10.1007/s00056-021-00279-1 33765157 10.1007/s00056-021-00279-1

[25] Sameshima GT, Sinclair PM (2001) Predicting and preventing root resorption: Part I. diagnostic factors. Am J Orthod Dentofacial Orthop 119:505–510. 10.1067/mod.2001.113409 11343022 10.1067/mod.2001.113409

[26] Wolford LM (2020) Comprehensive post orthognathic surgery orthodontics: complications, misconceptions, and management. Oral Maxillofac Surg Clin North Am 32:135–51. 10.1016/j.coms.2019.09.003 31685347 10.1016/j.coms.2019.09.003

[27] Özsoy ÖP, Değirmenci Z (2008) Ortodontik Tedavi Sırasında Görülen Kök Rezorpsiyonu’nun İncelenmesi. ADO Klin Bilim Derg 2:82–86

[28] Uribe FA, Farrell B (2019) Surgery-first approach in the orthognathic patient. Oral Maxillofac Surg Clin North Am 32:89–103. 10.1016/j.coms.2019.08.009 31685343 10.1016/j.coms.2019.08.009

[29] Marques LS, Chaves KCT, Rey AC, Pereira LJ, de Oliveira Ruellas AC (2011) Severe root resorption and orthodontic treatment: clinical implications after 25 years of follow-up. Am J Orthod Dentofacial Orthop 139:166–9. 10.1016/j.ajodo.2009.05.032 10.1016/j.ajodo.2009.05.032 21435537

[30] Castro IO, Alencar AH, Valladares-Neto J, Estrela C (2013) Apical root resorption due to orthodontic treatment detected by cone beam computed tomography. Angle Orthod 83:196–203. 10.2319/032112-240.1 22812378 10.2319/032112-240.1 PMC8793664

[31] Luo N, Chen Y, Li L, Wu Y, Dai H, Zhou J (2024) Multivariate analysis of alveolar bone dehiscence and fenestration in anterior teeth after orthodontic treatment: a retrospective study. Orthod Craniofac Res 27:287–296. 10.1111/ocr.12726 37929647 10.1111/ocr.12726

[32] Fuhrmann R, Bücker A, Diedrich P (1995) Assessment of alveolar bone loss with high resolution computed tomography. J Periodontal Res 30:258–63. 10.1111/j.1600-0765.1995.tb02131.x 7562322 10.1111/j.1600-0765.1995.tb02131.x

[33] Dos Santos MC, Iwaki LCV, Valladares-Neto J, Inoue-Arai MS, Ramos AL (2021) Impact of orthognathic surgery on the prevalence of dehiscence in Class II and Class III surgical-orthodontic patients: a cone beam computed tomographic study. Angle Orthod 91:611–618. 10.2319/062720-590.1 33836070 10.2319/062720-590.1 PMC8376160

[34] Evangelista K, de Faria Vasconcelos K, Bumann A, Hirsch E, Nitka M, Silva MAG (2010) Dehiscence and fenestration in patients with Class I and Class II Division 1 malocclusion assessed with cone-beam computed tomography. Am J Orthod Dentofacial Orthop 138:133.e1-133.e7. 10.1016/j.ajodo.2010.02.021 20691344 10.1016/j.ajodo.2010.02.021

[35] Ma H, Li W, Xu L et al (2021) Morphometric evaluation of the alveolar bone around central incisors during surgical orthodontic treatment of high-angle skeletal class III malocclusion. Orthod Craniofac Res 24:87–95. 10.1111/ocr.12408 32615016 10.1111/ocr.12408

