# Supplementary material for: 3D assessment of root resorption and soft tissue adaptations in surgery-first vs. early orthognathic surgery: a randomized clinical trial
Source: Clin Oral Investig. 2026 Mar 30;30(4):160. doi: 10.1007/s00784-026-06846-3 (PMC13035882; doi:10.1007/s00784-026-06846-3)
Supplement: Supplementary file 1 — Supplementary Material 1 (DOCX 16.1 KB) [file 784_2026_6846_MOESM1_ESM.docx]

| Supplementary Table 1. Intergroup comparison of baseline (T0) cephalometric parameters | | | |
| --- | --- | --- | --- |
| **Parameters** | **SF** | **SE** | ***p* value** |
|  | **Mean ± SD** | **Mean ± SD** |  |
| ANB (°) | -4.85 ± 2.50 | -5.17 ± 3.20 | 0.784 |
| Wits (mm) | -10.71 ± 3.78 | -11.35 ± 3.72 | 0.679 |
| SN–GoGn (°) | 31.41 ± 5.27 | 31.39 ± 5.91 | 0.994 |
| U1–PP (°) | 119.2 ± 5.00 | 120.82 ± 4.51 | 0.431 |
| U1–NA (mm) | 7.83 ± 2.79 | 7.71 ± 3.51 | 0.929 |
| IMPA (°) | 81.52 ± 6.86 | 86.48 ± 7.89 | 0.114 |
| L1–NB (mm) | 7.83 ± 2.79 | 4.38 ± 2.80 | 0.868 |
| L1–APog (mm) | 6.57 ± 3.44 | 7.00 ± 3.80 | 0.794 |
| Overjet (mm) | -2.82 ± 2.04 | -3.60 ± 3.30 | 0.506 |
| An independent-samples t-test was used for intergroup comparisons. SF: surgery-first; SE: surgery-early; SD: standard deviation. Statistical significance was set at *p* < 0.05.  Cephalometric measurements: ANB (°), A point–nasion–B point angle; Wits (mm), Wits appraisal; SN–GoGn (°), sella–nasion to gonion–gnathion angle; U1–PP (°), upper incisor to palatal plane angle; U1–NA (mm), linear distance from the upper incisor to the NA line; IMPA (°), incisor mandibular plane angle; L1–NB (mm), linear distance from the lower incisor to the NB line; L1–APog (mm), linear distance from the lower incisor to the A–Pogonion line; Overjet (mm), sagittal distance between the upper and lower central incisors. | | | |
